# Identifying the Presence of Prostate Cancer in Individuals with PSA Levels <20 ng ml^−1^ Using Computational Data Extraction Analysis of High Dimensional Peripheral Blood Flow Cytometric Phenotyping Data

**DOI:** 10.3389/fimmu.2017.01771

**Published:** 2017-12-18

**Authors:** Georgina Cosma, Stéphanie E. McArdle, Stephen Reeder, Gemma A. Foulds, Simon Hood, Masood Khan, A. Graham Pockley

**Affiliations:** ^1^School of Science and Technology, Nottingham Trent University, Nottingham, United Kingdom; ^2^John van Geest Cancer Research Centre, School of Science and Technology, Nottingham Trent University, Nottingham, United Kingdom; ^3^University Hospitals of Leicester NHS Trust, Leicester, United Kingdom

**Keywords:** prostate cancer, predictive modeling, immunophenotyping data, flow cytometry, PSA level, computational analysis, genetic algorithm, machine learning

## Abstract

Determining whether an asymptomatic individual with Prostate-Specific Antigen (PSA) levels below 20 ng ml^−1^ has prostate cancer in the absence of definitive, biopsy-based evidence continues to present a significant challenge to clinicians who must decide whether such individuals with low PSA values have prostate cancer. Herein, we present an advanced computational data extraction approach which can identify the presence of prostate cancer in men with PSA levels <20 ng ml^−1^ on the basis of peripheral blood immune cell profiles that have been generated using multi-parameter flow cytometry. Statistical analysis of immune phenotyping datasets relating to the presence and prevalence of key leukocyte populations in the peripheral blood, as generated from individuals undergoing routine tests for prostate cancer (including tissue biopsy) using multi-parametric flow cytometric analysis, was unable to identify significant relationships between leukocyte population profiles and the presence of benign disease (no prostate cancer) or prostate cancer. By contrast, a Genetic Algorithm computational approach identified a subset of five flow cytometry features (*CD*8^+^*CD*45*RA*^−^*CD*27^−^*CD*28^−^ (*CD*8^+^ Effector Memory cells); *CD*4^+^*CD*45*RA*^−^*CD*27^−^*CD*28^−^ (*CD*4^+^ Terminally Differentiated Effector Memory Cells re-expressing CD45RA); *CD*3^−^*CD*19^+^ (B cells); *CD*3^+^*CD*56^+^*CD*8^+^*CD*4^+^ (NKT cells)) from a set of twenty features, which could potentially discriminate between benign disease and prostate cancer. These features were used to construct a prostate cancer prediction model using the k-Nearest-Neighbor classification algorithm. The proposed model, which takes as input the set of flow cytometry features, outperformed the predictive model which takes PSA values as input. Specifically, the flow cytometry-based model achieved Accuracy = 83.33%, AUC = 83.40%, and optimal ROC points of FPR = 16.13%, TPR = 82.93%, whereas the PSA-based model achieved Accuracy = 77.78%, AUC = 76.95%, and optimal ROC points of FPR = 29.03%, TPR = 82.93%. Combining PSA and flow cytometry predictors achieved Accuracy = 79.17%, AUC = 78.17% and optimal ROC points of FPR = 29.03%, TPR = 85.37%. The results demonstrate the value of computational intelligence-based approaches for interrogating immunophenotyping datasets and that combining peripheral blood phenotypic profiling with PSA levels improves diagnostic accuracy compared to using PSA test alone. These studies also demonstrate that the presence of cancer is reflected in changes in the peripheral blood immune phenotype profile which can be identified using computational analysis and interpretation of complex flow cytometry datasets.

## 1 Introduction

The introduction of the serum Prostate-Specific Antigen (PSA) level as a biomarker for the presence of prostate cancer in 1986 prompted a progressive global increase in the diagnosis, and earlier diagnosis of the disease. The fact that most men are now diagnosed with organ-confined disease enables intervention with curative intent. However, although the initial diagnosis of prostate cancer in most men is based on a PSA test and digital rectal examination (DRE) (1), the PSA test has been criticized for its poor diagnostic specificity (30%) (2). Further investigations are, therefore, indicated in the event of an elevated PSA or abnormal DRE. These include a transrectal ultrasound (TRUS)-guided prostate biopsy and subsequent examination and reporting by a pathologist. However, TRUS-guided prostate biopsies have a documented sensitivity of only 39–52% (3), and cancer detection rates of around 25% on initial biopsies (4), and 18–32% on repeated biopsies (5, 6). This approach is also costly and rarely detects prostate cancers that an elevated PSA and/or DRE cannot predict. Although TRUS is commonly used to guide a biopsy, it is not, therefore, recommended for routine screening. An alternative approach to the TRUS is the Transperineal Template Prostate Biopsy (TPTPB), and we have previously shown that TPTPB can identify clinically significant prostate cancer in 71/122 (58%) of men with raised PSA, despite two previous sets of negative TRUS biopsies (7). An important element of these findings was that 61% of the patients in whom prostate cancer was diagnosed had a Gleason grade score ≥7 (most which were in the anterior zone), thereby automatically placing them into the “intermediate” or “high-risk” categories when applying established risk stratification criteria (7). The capacity of the TPTPB to identify more clinically significant tumors at an earlier stage, therefore, suggests that it is a better diagnostic test for localized prostate cancer than the TRUS biopsy. Given the ability of the TPTPB to detect prostate cancer at significantly higher rates than TRUS biopsies (8–12), we questioned whether we should move away from TRUS biopsies to TPTPB and whether PSA is actually a more specific biomarker for prostate cancer detection than had been previously thought. To this end, we performed a prospective study which directly compared the diagnostic potential of the TRUS and TPTPB approaches in the same cohort of biopsy naïve men with an elevated PSA <20 ng ml^−1^ and a benign feeling prostate on a DRE. These patients, therefore, served as their own controls (13). The study demonstrated that the TRUS biopsy detected cancer in 32 versus 60% with TPTP, and that TPTPB is associated with a significantly higher prostate cancer detection rate than TRUS biopsies in biopsy naïve men with PSA <20 ng ml^−1^ and a benign feeling DRE (13). However, given that TRUS guided prostate biopsies are associated with a 5% risk of urosepsis (which can be life-threatening), and that TPTPB is performed under general anesthetic and associated with a 5% risk of urinary retention, both procedures are associated with a significant cost and potential for complications. It is also essential that men with low-risk prostate cancer are not diagnosed as having cancer, as they do not require any active treatment and such individuals are “labeled” as having cancer. This can have profound adverse psychological and financial consequences, and assign them to life-long surveillance. The fundamental aim of this study is, therefore, to develop an approach which delivers a high level of diagnostic accuracy for asymptomatic men with an elevated PSA <20 ng l^−1^. The development of such approaches will spare men with benign disease or low-risk cancer from unnecessary invasive diagnostic procedures such as TRUS-guided prostate biopsies or TPTPB. Given the reciprocal interactions between tumors and the immune system, we hypothesized that the presence of disease, disease recurrence, and therapeutic resistance may be influenced, reflected in, or predicted by tumor-related immunoregulatory events that can be identified by changes in immune phenotypes in the periphery. We, therefore, proposed that the analysis of immune phenotyping datasets using multi-parametric flow cytometric analysis can identify features that reflect the presence of disease and/or predict disease progression (14). Although flow cytometry provides a vital tool for exploring, explaining, and understanding complex cellular dynamics and processes in a variety of experimental and clinical settings (15), key challenges with multi-parametric flow cytometry include the analysis and interpretation of the complex and increasingly multidimensional data and its conversion into biologically and clinically useful information. This study attempts to address and resolve some of these challenges using computational intelligence methods. Computational intelligence methods comprise evolutionary algorithms (also known as metaheuristic optimization, or nature-inspired optimization algorithms) coupled with machine learning methods, and hybrids of these. A type of machine learning method, supervised learning, is used to derive prediction models which can be very effective in dealing with uncertainty, noise, and dimensionality in data. Supervised learning methods can learn from existing data to make informed predictions using new patient data, and have been widely adopted for prostate cancer prediction tasks when using clinical and biomedical data (16). It is now time to embrace computational intelligence methods for the analysis of flow cytometry data, since statistical methods alone may not be sufficient for the task of analyzing and modeling such complex data (16). Herein, we assess whether advanced computational analysis of peripheral blood flow cytometry immunophenotyping data from a selected cohort of individuals can generate prediction models with potential clinical value and identify the presence of prostate cancer in asymptomatic individuals with a PSA level <20 ng ml^−1^. The computational models and algorithms are trained to make predictions on new and previously unseen data using existing data. Significantly, this approach has identified a novel prostate cancer immunophenotyping “fingerprint” which could potentially be used to identify the presence of prostate cancer in asymptomatic men having PSA levels <20 ng ml^−1^; and which outperforms the predictive value of the PSA test alone. We have also shown that combining flow cytometry predictors with PSA levels improves diagnostic accuracy. Taken together, these studies demonstrate that the presence of cancer is reflected in changes in the peripheral blood immune phenotype profile which can be identified using computational analysis and interpretation of complex flow cytometry datasets, and the value of computational intelligence-based approaches for interrogating immunophenotyping datasets.

## 2 Materials

### 2.1 Data Collection

Patients with suspected prostate cancer attending the Urology Clinic at Leicester General Hospital (University Hospitals of Leicester NHS Trust, Leicester, UK) were examined by Professor Masood Khan (Consultant Urologist) and Mr. Shady Nafie (Registrar in Urology). Samples were obtained from a selected cohort of patients which met the following criteria—being biopsy naïve, with a PSA level of <20 ng ml^−1^ and agreeing to undergo simultaneous TRUS biopsy (12 cores) and a transperineal template prostate biopsy (TPTPB) (36 cores) procedures under general anesthetic. Samples from the TPTPB cohort were collected from 24 October 2012 to 15 August 2014. Further details on how patients were recruited and treated are described in Nafie et al. (7). The cohort comprised samples from 72 males who had a TRUS-guided biopsy and then a TPTPB. The mean age for this cohort was 66 years old (age range of 50–84 years old). Given the more definitive diagnostic power of the TPTPB (7, 13), samples that were considered as being from individuals with benign disease were obtained from this cohort. A total of 41 (56.94%) patients were diagnosed with prostate cancer. The remaining 31 (43.06%) patients were classed as having benign disease following pathological examination and the application of established criteria. Of those patients diagnosed with benign disease, 10 patients were diagnosed with High Prostatic Intraepithelial Neoplasia (High-Grade); 10 patients were diagnosed with Atypical Small Acinar Proliferation and 2 patients with Atypia. The remaining 9 patients were diagnosed as having benign disease. Patients with multi-focal high-grade PIN or ASAP commonly have a prostatic core biopsy showing a focus which is suspicious for, but not diagnostic of, cancer (17).

### 2.2 Ethics Statement

Research Protocols were registered and approved by the National Research Ethics Service (NRES) Committee East Midlands and by the Research and Development Department in the University Hospitals of Leicester NHS Trust. All participants were given information sheets explaining the nature of the study and all provided informed consent. All samples were collected by suitably qualified individuals using standard procedures. Ethical approval for the collection and use of samples from the TPTPB cohort (Project Title: Defining the role of Transperineal Template-guided prostate biopsy) was given by NRES Committee East Midlands-Derby 1 (NREC Reference number: 11*/EM/*3012; UHL11068). Ethical approval for the collection of peripheral blood from healthy volunteers was obtained from the Nottingham Trent University College of Science and Technology Human Ethics Committee (Application numbers 165 and 412).

### 2.3 Flow Cytometric Analysis

Peripheral blood (60 ml) was collected from all patients using standard clinical procedures. Aliquots (30 ml) were transferred into two sterile 50 ml polypropylene (Falcon) tubes containing 300 µl of sterilized Heparin (1000 U ml^−1^, Sigma). Anti-coagulated samples were immediately transferred to the John van Geest Cancer Research Centre at Nottingham Trent University (Nottingham, UK) and were processed immediately upon receipt (as described in this section), and within 3 h of collection. 200 µl of whole blood was used to profile the key immune cell subsets in the periphery (Overview of the Immune System: “OVIS”—see Table 1).

**Table 1:** Monoclonal antibody panel.

Antibody	Fluorochrome	Clone No.	Supplier
CD8	FITC	SK1	Biolegend
CD19	PE	HIB19	Biolegend
CD28	PE-Texas Red (ECD)	CD28.2	Beckman Coulter
CD56	PE-Cy5	NCAM	Biolegend
CD3	PE-Cy7	HIT3a	Biolegend
CD45RA	Allophycocyanine (APC)	HI100	eBioscience
CD14	Alexa Fluor 700	HCD14	Biolegend
CD27	APC eFluor 780	O323	eBioscience
CD45	Pacific Blue	J33	Beckman Coulter
CD4	Krome Orange	13B8.2	Beckman Coulter

**Figure 1:**
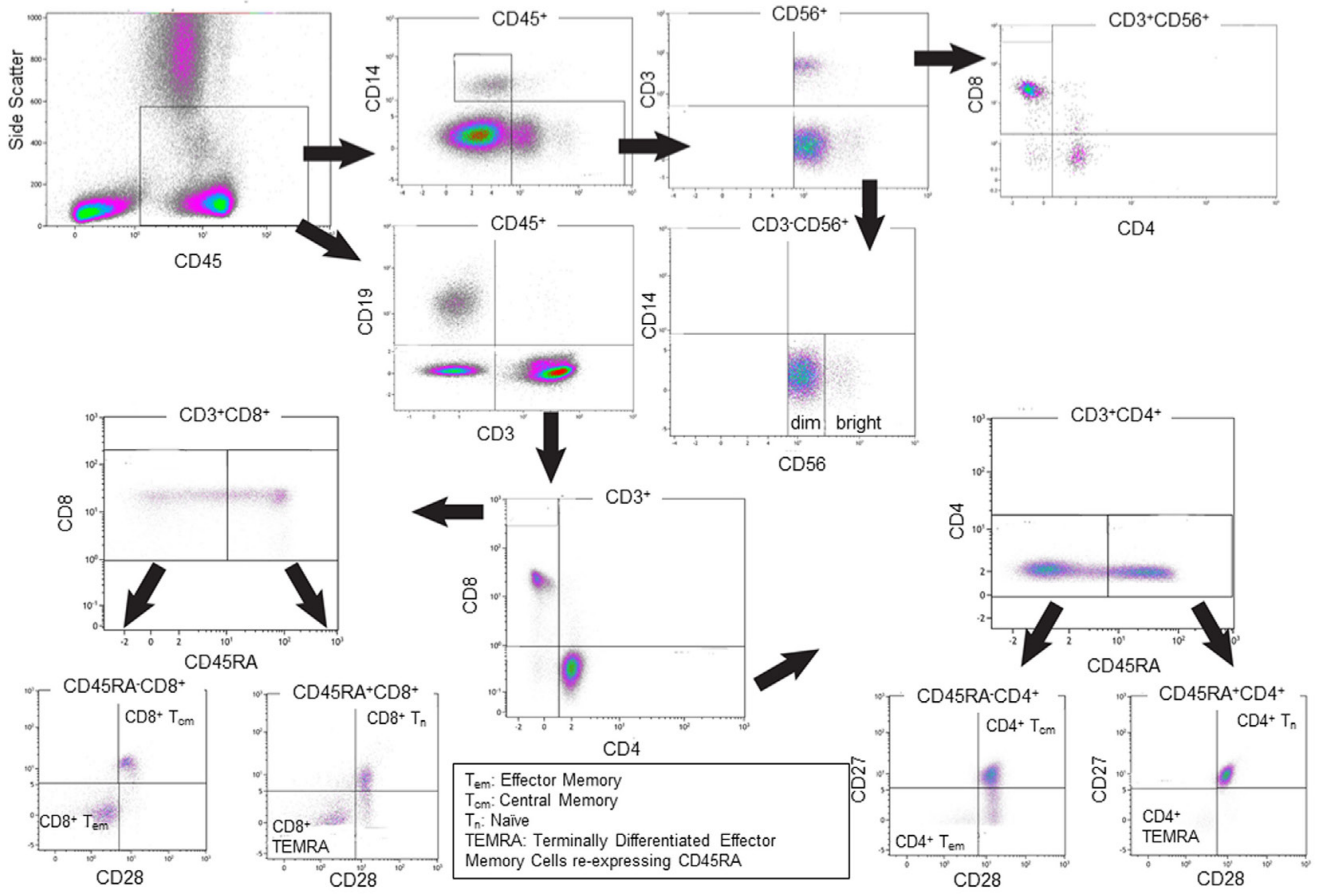
Representative gating strategies for the flow cytometric analysis of single cells. The Overview of the Immune System (OVIS) staining panel confirmed *CD*45 expression then determined cell populations as *CD*14^+^ monocytes, *CD*3^−^*CD*56^+^ NK cells (with *CD*56*^bright^* and *CD*56*^dim^* subsets), *CD*3^+^*CD*56^+^ NKT cell subpopulations, *CD*19^+^ B cells, *CD*3^+^*CD*4^+^ and *CD*3^+^*CD*8^+^ Naïve, Central Memory, Effector Memory, Terminally Differentiated Effector Memory Cells Expressing CD45RA T cells populations. The definition of monocytes based on *CD*45^+^*CD*4^+^ generated the same data as defining them based on *CD*3^−^*CD*14^+^ (data not shown).

Absolute cell counts in whole blood samples were determined by the inclusion of BD Trucount™ beads (BD Biosciences; Mountain View, CA, USA), as per the manufacturer’s protocol. For the flow cytometric analysis, 100 µl of blood was mixed directly in the BD Trucount™ bead tube and T cell, B cell, and NK cell populations identified using the conjugated monoclonal antibodies (mAbs) detailed in Table 1. For the staining, cells were incubated for 15 min at room temperature, protected from the light, after which erythrocytes were lysed by incubating samples for 15 min at room temperature in BD Pharm Lyse™ (BD Biosciences). Once staining was complete, cells were washed in phosphate buffered saline (PBS), resuspended in Coulter Isoton™ diluent. Data were acquired within 1 h using a 10-color/3-laser Beckman Coulter Gallios™ flow cytometer and analyzed using Kaluza™ v1.3 data acquisition and analysis software (Beckman Coulter). Controls used a “Fluorescence minus One,” “FMO” approach. A typical gating strategy for the analyses is presented in Figure 1.

### 2.4 Data Normalization and Statistical Analysis

For this study, we considered a feature to be the grouped set of flow cytometry phenotypic variables shown in Table 2. The mean and Standard Deviation (SD) values of each flow cytometry feature shown in Table 2 indicate clear variation, as a consequence of which data were normalized to put them on the same scale and enable the comparison of two or more variables (i.e., flow cytometry features). Let *X_m_*_x_*_n_* = [*x_ij_*] be a m x n matrix with *m* rows and *n* columns. Z-score normalization was applied to each column *n* of matrix *X*. Applying normalization returned the z-score value for each matrix element *x_ij_*, and each column *j* of matrix *X* was centered to have a mean value of *0* and scaled to have a SD value of 1. The standardized data set retains the shape properties of the original data set (same skewness and kurtosis). The z-score normalization function is shown in Function (1):

(1) $$z=\frac{\left(x_{ij}-\bar{x}\right)}{\sigma }$$

where *x_ij_* is a data point; $\bar{x}$ is the mean value of column *j*; *σ* is the SD; and *z* is the transformed value of data point *x_ij_*.

**Table 2:** Flow Cytometry features.

Feature ID	Flow cytometry feature	Mean	SD
1	*CD*3^+^*CD*8^+^	450.39	402.03
2	*CD*8^+^*CD*45*RA^+^CD*27^+^*CD*28^+^	96.92	75.99
3	*CD*8^+^*CD*45*RA*^−^*CD*27^+^*CD*28^−^	68.45	58.73
4	*CD*8^+^*CD*45*RA*^−^*CD*27^−^*CD*28^−^	45.37	104.69
5	*CD*8^+^*CD*45*RA^+^CD*27^−^*CD*28^−^	120.05	197.85
6	*CD*3^+^*CD*4^+^	877.88	468.35
7	*CD*4^+^*CD*45*RA^+^CD*27^+^*CD*28^+^	393.72	214.27
8	*CD*4^+^*CD*45*RA*^−^*CD*27^+^*CD*28^+^	311.24	211.16
9	*CD*4^+^*CD*45*RA*^−^*CD*27^−^*CD*28^−^	17.78	39.78
10	*CD*4^+^*CD*45*RA^+^CD*27^−^*CD*28^−^	14.19	35.48
11	*CD*45^+^*CD*14^+^	116.16	87.19
12	*CD*3^−^*CD*19^+^	257.70	251.40
13	*CD*3^+^*CD*56^+^*NKT*	76.54	85.74
14	*CD*3^−^*CD*56^+^*NK*	260.34	202.84
15	*CD*3^−^*CD*56*^low^*	253.20	192.27
16	*CD*3^−^*CD*56*^high^*	16.06	14.66
17	*CD*3^+^*CD*56^+^*CD*8^+^*CD*4^+^	5.67	16.16
18	*CD*3^+^*CD*56^+^*CD*8^+^*CD*4^−^	53.32	59.76
19	*CD*3^+^*CD*56^+^*CD*8^−^*CD*4^+^	10.96	20.41
20	*CD*3^+^*CD*56^+^*CD*8^−^*CD*4^−^	6.59	6.58

*Mean and Standard Deviation (SD) values of raw data*.

**Figure 2:**
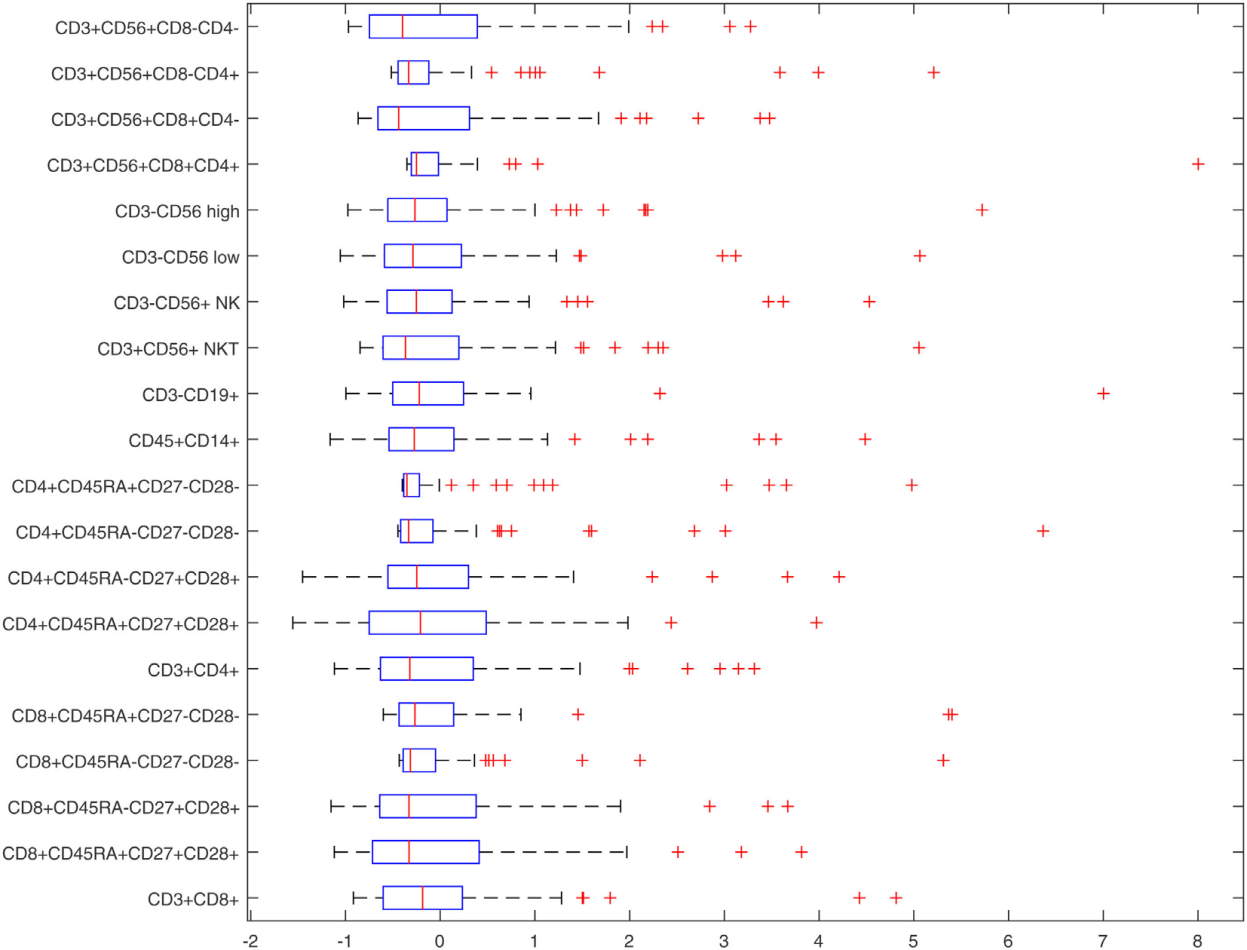
Box-plots of normalized flow cytometry features.

**Figure 3:**
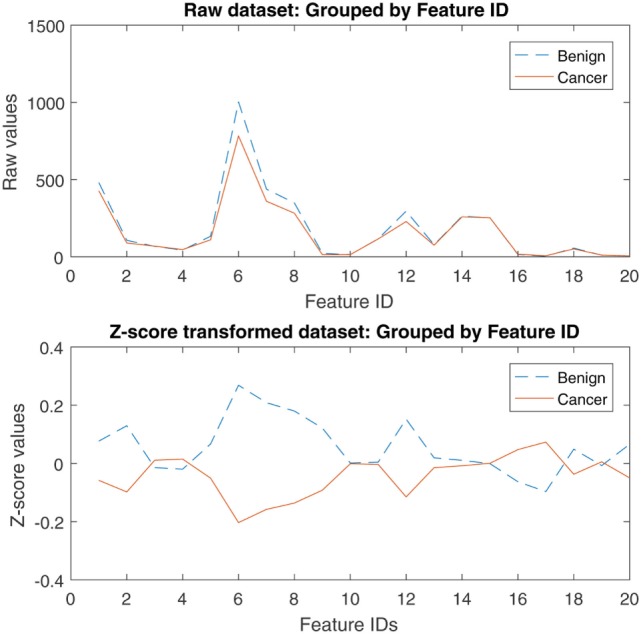
Raw and z-score transformed values of the flow cytometry variables derived from individuals with benign disease and patients with prostate cancer.

Figure 2 illustrates the distribution of the flow cytometry features in the form of box plots, and allows for quick visualization of variability. Outliers were included in the analyses as it is important to consider those “out of range values” when creating a prediction model. Figure 3 illustrates the flow cytometry values derived from individuals with benign disease and patients with prostate cancer before and after data normalization.

Table 3 provides descriptive statistics of the normalized dataset, and these are also illustrated in Figure 2. The Interquartile range (IQR) is an informative measure of variability and determined by computing the distance between the Upper Quartile (i.e., top) and Lower Quartile (i.e., bottom) of the box. The features with the smallest degree of variability are those with the smallest IQR values, and hence: *CD*4^+^*CD*45*RA^+^CD*27^−^*CD*28^−^ (ID 10, first smallest); *CD*3^+^*CD*56^+^*CD*8^+^*CD*4^+^ (ID 17, second smallest); *CD*3^+^*CD*56^+^*CD*8^−^*CD*4^+^ (ID 19, third smallest); *CD*4^+^*CD*45*RA*^−^*CD*27^−^*CD*28^−^ (ID 9, fourth smallest), *CD*8^+^*CD*45*RA*^−^*CD*27^−^*CD*28^−^ (ID 4, fifth smallest). These variables, therefore, appear to the best candidate predictors when considered independently.

**Table 3:** Descriptive statistics of the normalized dataset.

	Flow cytometry feature	Range	Minimum	Maximum	IQR	Skewness
1	*CD*3^+^*CD*8^+^	5.73	−0.92	4.81	0.84	2.92
2	*CD*8^+^*CD*45*RA^+^CD*27^+^*CD*28^+^	4.93	−1.12	3.82	1.13	1.65
3	*CD*8^+^*CD*45*RA*^−^*CD*27^+^*CD*28^+^	4.82	−1.15	3.67	1.02	1.74
4	*CD*8^+^*CD*45*RA*^−^*CD*27^−^*CD*28^−^	5.75	−0.43	5.31	0.34	4.41
5	*CD*8^+^*CD*45*RA^+^CD*27^−^*CD*28^−^	6.00	−0.60	5.40	0.58	4.44
6	*CD*3^+^*CD*4^+^	4.43	−1.12	3.32	0.98	1.71
7	*CD*4^+^*CD*45*RA^+^CD*27^+^*CD*28^+^	5.53	−1.56	3.97	1.23	1.27
8	*CD*4^+^*CD*45*RA*^−^*CD*27^+^*CD*28^+^	5.66	−1.45	4.21	0.85	2.17
9	*CD*4^+^*CD*45*RA*^−^*CD*27^−^*CD*28^−^	6.81	−0.45	6.36	0.34	4.41
10	*CD*4^+^*CD*45*RA^+^CD*27^−^*CD*28^−^	5.38	−0.40	4.98	0.17	3.46
11	*CD*45^+^*CD*14^+^	5.65	−1.16	4.49	0.68	2.65
12	*CD*3^−^*CD*19^+^	8.00	−1.00	7.00	0.75	5.02
13	*CD*3^+^*CD*56^+^*NKT*	5.90	−0.85	5.05	0.80	2.46
14	*CD*3^−^*CD*56^+^*NK*	5.55	−1.02	4.53	0.69	2.62
15	*CD*3^−^*CD*56*^low^*	6.12	−1.06	5.06	0.81	2.67
16	*CD*3^−^*CD*56*^high^*	6.70	−0.97	5.72	0.63	3.18
17	*CD*3^+^*CD*56^+^*CD*8^+^*CD*4^+^	8.35	−0.35	8.00	0.29	7.28
18	*CD*3^+^*CD*56^+^*CD*8^+^*CD*4^−^	4.34	−0.87	3.48	0.97	1.80
19	*CD*3^+^*CD*56^+^*CD*8^−^*CD*4^+^	5.73	−0.52	5.21	0.32	3.64
20	*CD*3^+^*CD*56^+^*CD*8^−^*CD*4^−^	4.25	−0.97	3.28	1.14	1.48

**Figure 4:**
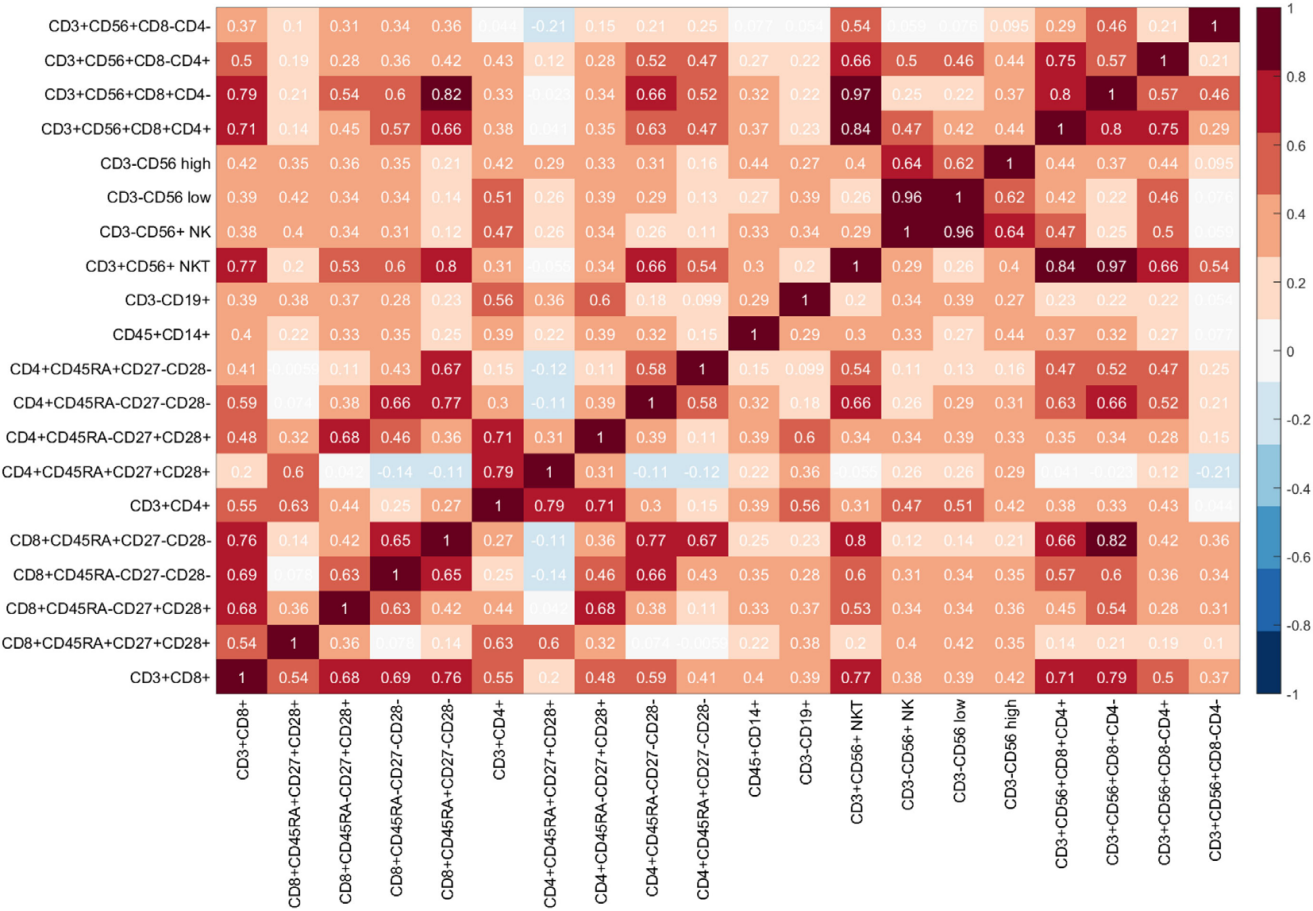
Heatmap of flow cytometry features: Each cell of the heatmap provides a Spearman rho correlation value between two flow cytometry features.

The Kruskal–Wallis test (“one-way ANOVA on ranks”) tested for statistically significant differences between the mean ranks of the normalized flow cytometry variables observed in individuals with benign disease and patients with prostate cancer due to the presence of unequal variances, and demonstrated there to be no statistically significant differences at the alpha level of *α* = 0.05 in the mean ranks of the flow cytometry features between these two groups (Table 4). A more sophisticated approach that has the potential to determine which features would better indicate the presence of disease was, therefore, adopted. For this, a Genetic Algorithm was used to explore the different combinations of features and return the optimal combination of features which indicate the presence of prostate cancer. As a final stage of the analysis, and prior to applying a Genetic Algorithm for feature selection, it is useful to determine whether any correlations among the flow cytometry features exist. For this, the non-parametric Spearman rank correlation assessed the degree of association between flow cytometry features. The rho values arising from this analysis were plotted in a heatmap graph (shown in Figure 4) in order to visualize those feature pairs having strong positive and strong negative correlations. Figure 4 shows that many pairs have positive correlation values (color red). The *p* values were computed to determine which of these correlations were significant at *α* = 0.05. The rho correlation values range from −1.0 to +1.0. A value of 0 suggests no correlation, a value of +1.0 suggests a strong positive correlation and a value of −1.0 suggests a strong negative correlation. A total of 141 unique pairs of features returned significant correlations with *p* < 0.05.

**Table 4:** Results of the Kruskal–Wallis test for testing for significant differences, at α < 0.05, between the mean ranks of the normalized flow cytometry variables observed between patients with benign disease and patients with prostate cancer.

	Flow cytometry feature	Chi-Sq. *χ^2^*	Asy. Sig. p value
1	*CD*3^+^*CD*8^+^	1.73	0.19
2	*CD*8^+^*CD*45*RA^+^CD*27^+^*CD*28^+^	0.82	0.37
3	*CD*8^+^*CD*45*RA*^−^*CD*27^+^*CD*28^+^	0.04	0.83
4	*CD*8^+^*CD*45*RA*^−^*CD*27^−^*CD*28^−^	0.06	0.81
5	*CD*8^+^*CD*45*RA^+^CD*27^−^*CD*28^−^	0.44	0.51
6	*CD*3^+^*CD*4^+^	3.72	0.05
7	*CD*4^+^*CD*45*RA^+^CD*27^+^*CD*28^+^	1.33	0.25
8	*CD*4^+^*CD*45*RA*^−^*CD*27^+^*CD*28^+^	1.79	0.18
9	*CD*4^+^*CD*45*RA*^−^*CD*27^−^*CD*28^−^	3.44	0.06
10	*CD*4^+^*CD*45*RA^+^CD*27^−^*CD*28^−^	0.88	0.35
11	*CD*45^+^*CD*14^+^	0.80	0.37
12	*CD*3^−^*CD*19^+^	0.74	0.39
13	*CD*3^+^*CD*56^+^*NKT*	0.59	0.44
14	*CD*3^−^*CD*56^+^*NK*	0.74	0.39
15	*CD*3^−^*CD*56*^low^*	0.96	0.33
16	*CD3*^−^*CD56^high^*	0.52	0.47
17	*CD*3^+^*CD*56^+^*CD*8^+^*CD*4^+^	0.61	0.44
18	*CD*3^+^*CD*56^+^*CD*8^+^*CD*4^−^	0.68	0.41
19	*CD*3^+^*CD*56^+^*CD*8^−^*CD*4^+^	2.85	0.09
20	*CD*3^+^*CD*56^+^*CD*8^−^*CD*4^−^	0.03	0.86

The large number of pairs having significant correlations presents significant challenges for identifying features which better identify the presence of disease. This is because if two features have a strong correlation, then only one of those features should be selected as a candidate predictor. A Genetic Algorithm evaluates these combinations and identifies those features that, as a combination, deliver the best subset of predictors.

## 3 Results

### 3.1 Experiment Methodology

The aim of the experiments is to identify a suitable set of features which would, as a combination, deliver an immunophenotypic “fingerprint” for determining whether an individual with Prostate-Specific Antigen (PSA) levels below 20 ng ml^−1^ has prostate cancer in the absence of definitive biopsy-based evidence. This fingerprint, or set of features, would then be utilized to construct a prediction model. Given that the optimum number of features was unknown, a Genetic Algorithm (18) was applied *λ* times, with *λ* = 2, 3, …, *n* where *n* is the total number of flow cytometry features. Therefore, each time the Genetic Algorithm was run a combination containing *λ* number of features was returned. A total of 19 subsets of features were returned by the Genetic Algorithm, with the first subset *s*_1_ containing the best 2 selected features; subset *s*_2_ the best 3 selected features, subset *s*_3_ the best 4 selected features, and so forth. Each subset, *s_i_* of selected features, was input into a kNN classifier. Experiments were conducted with kNN using various distance measures, as this would allow for it to be tuned for the specific problem at hand. The number of kNN neighbors was set to *k* = 2 and was chosen experimentally to be the best setting. The state-of-the-art Leave-One-Out Cross Validation (LOOCV) approach was adopted for evaluating the performance of the kNN classifier using various parameter settings. During LOOCV, the training and testing process is repeated m times and in every iteration, a different patient record is left out for testing until all records are left out (19). To perform the evaluations, the actual outputs returned by the classification model during the validation stage were compared against the targets (i.e., known outputs). The Receiver Operating Characteristic (ROC) curves were created and the optimal cut-off points (optimal ROC point (ORP): False Positive Rate (FPR), True Positive Rate (TPR)) were computed with the alpha value set to *α* = 0.05 (95% Confidence Interval). An efficient classification system (i.e., prediction model) would return the largest Area Under the Curve (AUC); a high number of True Positives; and a low number of False Positives. The methods of Hanley and McNeil (20, 21) were used for the calculation of the Standard Error of an Area Under the Curve (AUC (SE)), and the Binomial Exact Confidence Interval for an Area Under the Curve (AUC (BEC)) was also calculated.

### 3.2 Prostate Cancer Prediction Using Immunophenotyping Data

This section discusses the results of the experiments when tuning the kNN with various distance measures and when using each subset, *s_i_*, of flow cytometry features which were returned by the Genetic Algorithm. Table 5 shows the best results that were achieved after applying the kNN classifier using each subset of features and different distance measures. As shown in Table 5, the best performance was achieved using the FC-PM(Correlation(5)) which reached an AUC = 83.40% and Optimal ROC point of FPR = 16.13%, TPR = 82.93%. The FC-PM(Correlation(5)) utilized 5 flow cytometry features with IDs: 4, 9, 10, 12, 17 which correspond to flow cytometry features: *CD*8^+^*CD*45*RA*^−^*CD*27^−^*CD*28^−^ (*CD*8^+^ Effector Memory cells); *CD*4^+^*CD*45*RA*^−^*CD*27^−^*CD*28^−^ (*CD*4^+^ Effector Memory Cells); *CD*4^+^*CD*45*RA^+^CD*27^−^*CD*28^−^ (*CD*4^+^ Terminally Differentiated Effector Memory Cells re-expressing CD45RA); *CD*3^−^*CD*19^+^ (B cells); *CD*3^+^*CD*56^+^*CD*8^+^*CD*4^+^ (NKT cells).

**Table 5:** FC-based prediction models using kNN classification and the selected flow cytometry features.

Prediction model name	Feature IDS	Accuracy (%)	AUC (%)	Optimal ROC Point (%)	
				FPR	TPR
FC-PM(Correlation(5))	4, 9, 10, 12, 17	83.33	83.40	16.13	82.93
FC-PM(Cosine(6))	4, 9, 10, 11, 12, 17	83.33	81.83	29.03	92.68
FC-PM(Chebychev(6))	4, 9, 10, 11, 12, 17	81.94	81.39	22.58	85.37
FC-PM(Minkowski(6))	4, 9, 10, 11, 12, 17	80.56	79.78	25.81	85.37
FC-PM(Euclidean(6))	4, 9, 10, 11, 12, 17	80.56	79.78	25.81	85.37
FC-PM(Seuclidean(6))	4, 9, 10, 11, 12, 17	80.56	79.78	25.81	85.37
FC-PM(Mahalanobis(6))	4, 9, 10, 11, 12, 17	77.78	76.55	32.26	85.37
FC-PM(Cityblock(7))	4, 9, 10, 11, 12, 16, 17	77.78	76.55	32.26	85.37
FC-PM(Spearman(8))	2, 4, 9, 10, 11, 12, 17, 19	83.33	70.89	38.71	80.49

*The feature IDs map those presented in Table 2. The naming of the models includes the distance measure and number of features which were selected by the GA*.

**Figure 5:**
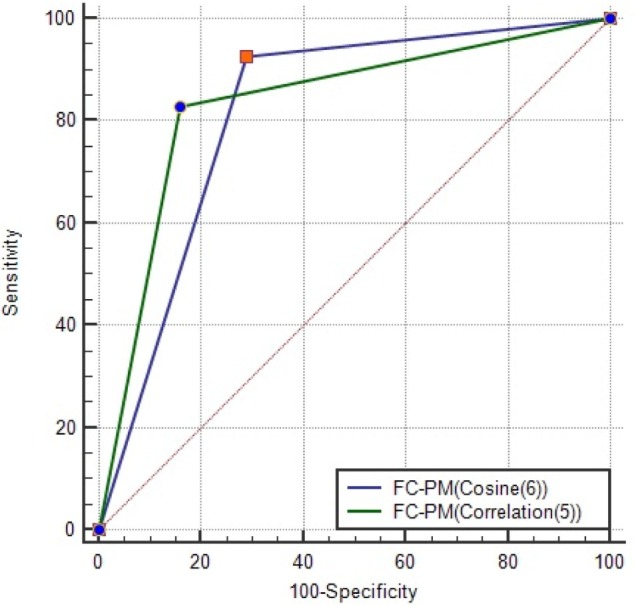
AUC for FC-PM(Cosine(6)) and FC-PM(Correlation(5)). The ORP of each model is also shown on the graph. FC-PM (Cosine(6)) has ORP (TPR = 92.68, FPR = 29.03) and FC-PM (Correlation (5)) has ORP (TPR = 82.93, FPR = 16.13).

Given that this set contains the best combination of flow cytometry predictors, it can be used as a signature for distinguishing between the presence of benign disease and cancer. FC-PM(Cosine(6)) achieved the same value for Accuracy as FC-PM(Correlation(5)) using 6 features. Feature *CD*45^+^*CD*14^+^ (ID 11) was included in the feature set used by FC-PM(Cosine(6)). Figure 5 shows the AUCs and optimal ROC Points of the two flow cytometry-based prediction models, FC-PM(Correlation(5)) and FC-PM(Cosine(6)). FC-PM (Cosine(6)) achieved a 12.9% higher False Positive Rate than FC-PM (Correlation(5)) (Table 5), and lower Confidence Interval(CI) values shown in Table 6, which suggests that it has weaker ability than FC-PM(Correlation(5)) to discriminate between benign and cancer patients. In addition, Table 6 shows the percentage of patients correctly classified in each group. FC-PM(Cosine(6)) achieved a lower predictive accuracy for benign patients compared to FC-PM(Correlation(5)), but correctly classified more cancer patients. The comparison suggests that FC-PM(Cosine(6)) is relatively more likely to misclassify benign patients as cancer patients, which is not a desirable outcome, and thus the model’s confidence in identifying benign disease is lower.

**Table 6:** A comparison using FC-PM(Correlation(5)) and FC-PM(Cosine(6)).

	FC-PM(Correlation(5))	FC-PM(Cosine(6))
AUC%	83.40	81.83
AUC (SE)^a^	0.0514	0.0550
AUC 95% CI^b^	0.728–0.911	0.710–0.899
Benign (% of correctly classified)	83.87	70.97
Cancer (% of correctly classified)	82.93	92.68
Misclassified (%)	16.67	16.67

*^a^Hanley and McNeil (20)*.

*^b^Binomial exact*.

Revisiting the results which are presented in Table 3, features *CD*8^+^*CD*45*RA*^−^*CD*27^−^*CD*28^−^ (ID 4); *CD*4^+^*CD*45*RA*^−^*CD*27^−^*CD*28^−^ (ID 9); *CD*4^+^*CD*45*RA^+^CD*27^−^*CD*28^−^ (ID 10); *CD*3^+^*CD*56^+^*CD*8^+^*CD*4^+^ (ID 17) were among those flow cytometry features with the smallest IQR values (and, therefore, least variability in data) and which would potentially be good candidates for indicating the presence cancer. Furthermore, the Genetic Algorithm identified an additional flow cytometry feature as part of the selected features (*CD*3^−^*CD*19^+^ (ID 12)) which was not an obvious candidate during the initial statistical analysis. When feature ID12 is placed into a group with other features, it contributes to improving prediction performance. This reinforces the point as to why it is important to examine combinations of features rather than individual features when choosing those which would make a cancer predictors (i.e., fingerprint). Importantly, not all flow cytometry features with a low IQR are needed to reach high predictive accuracy, and a subset containing the optimal combination of features was created using the Genetic Algorithm.

The heatmap in Figure 4 shows that the correlation values between the five selected features range from +0.10 to +0.66, with six out of the ten pairs having a weak correlation value *rho* < 0.50 (ID 4, ID 10) = 0.43, (ID 4, ID 12) = 0.28, (ID 9, ID 12) = 0.18, (ID 10,ID 12) = 0.10, (ID 10, ID 17) = 0.47, (ID 12, ID 17) = 0.23 and the remaining four pairs having moderate correlation values (ID 4, ID 9) = 0.66, (ID 4, ID 17) = 0.57, (ID 9, ID 10) = 0.58, and (ID 9, ID 17) = 0.63, thereby suggesting that these five features are most suitable, since none of these pairs are highly correlated. Hence, we can conclude that the flow cytometry features: *CD*8^+^*CD*45*RA*^−^*CD*27^−^*CD*28^−^ (*CD*8^+^ Effector Memory cells); *CD*4^+^*CD*45*RA*^−^*CD*27^−^*CD*28^−^ (*CD*4^+^ Effector Memory Cells); *CD*4^+^*CD*45*RA^+^CD*27^−^*CD*28^−^ (*CD*4^+^ Terminally Differentiated Effector Memory Cells re-expressing CD45RA); *CD*3^−^*CD*19^+^ (B cells); *CD*3^+^*CD*56^+^*CD*8^+^*CD*4^+^ (NKT cells) can be considered as an immunophenotyping profile which predicts the presence of prostate cancer in men with Prostate-Specific Antigen (PSA) levels below 20 ng ml^−1^.

### 3.3 Prostate Cancer Prediction: Immunophenotyping versus Prostate-Specific Antigen (PSA) Data

**Figure 6:**
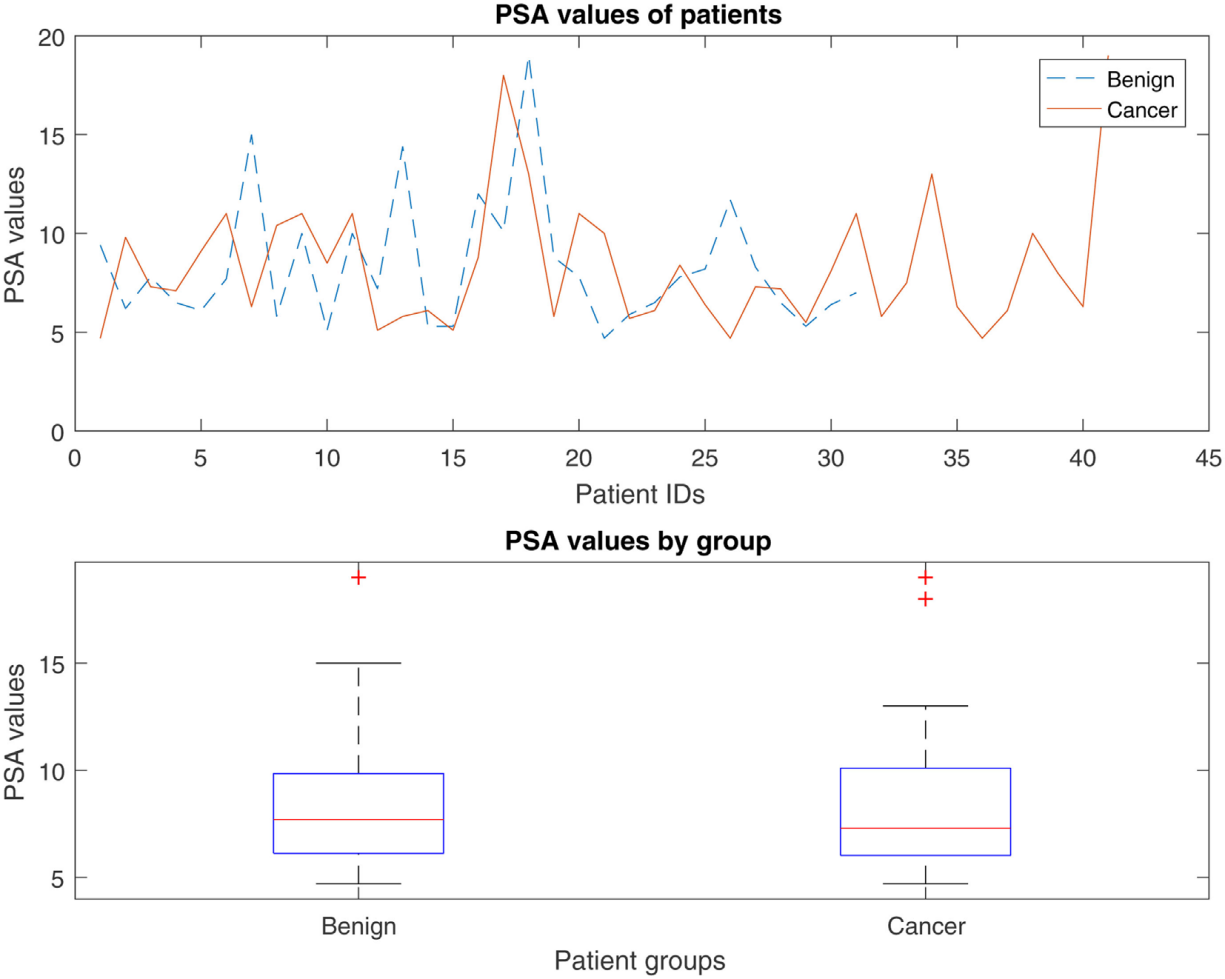
Distribution of the PSA values for individuals with benign disease and patients with prostate cancer.

The Prostate-Specific Antigen (PSA) test measures circulating levels of PSA and is currently considered to be the best method for identifying an increased risk of localized prostate cancer. However, elevated PSA levels do not necessarily indicate the presence of prostate cancer, and a normal PSA test does not necessarily exclude the presence of prostate cancer. PSA values tend to rise with age, and the total PSA levels (ng ml^−1^) recommended by the Prostate Cancer Risk Management Programme are as follows (22): 50–59 years, *PSA* ≥ 3.0; 60–69 years, *PSA* ≥ 4.0; and 70 and over, *PSA* > *5.0*. According to a study by the European Study of Screening for Prostate Cancer, screening can significantly reduce death from prostate cancer by 29% (23–25). Herein, we compare the capacity of the proposed flow cytometry-based prostate cancer predictive model (FC-PM) and a predictive model based on PSA blood test results (PSA-PM) to discriminate between benign disease and prostate cancer. Since PSA values were already between 1 and 20, it was not necessary to apply z-score transformation. Figure 6 shows the PSA values for individuals with benign disease and patients with cancer. A Kruskal–Wallis test sought significant differences between the mean rank PSA values of the benign disease and cancer groups. The test indicated that there were no significant differences in the mean rank PSA values between the individuals with benign disease and patients with cancer, *χ*^2^ (1, *N* = 72) = 0.03, *p* = 0.955.

PSA values were input into the kNN model and performance was evaluated using the LOOCV approach. Although experiments were performed with various distance measures, the Cityblock, Mahalanobis, Minkowski, Seuclidean, Euclidean and Chebychev returned exactly the same results, as shown in Table 7.

**Table 7:** Prediction using PSA data as input into the kNN classification model.

Prediction model	Accuracy (%)	AUC (%)	Optimal ROC Point (%)	
			FPR	TPR
PSA-PM(Cityblock)	77.78	76.95	29.03	82.93
PSA-PM(Mahalanobis)	77.78	76.95	29.03	82.93
PSA-PM(Minkowski)	77.78	76.95	29.03	82.93
PSA-PM(Seuclidean)	77.78	76.95	29.03	82.93
PSA-PM(Euclidean)	77.78	76.95	29.03	82.93
PSA-PM(Chebychev)	77.78	76.95	29.03	82.93
PSA-PM(Correlation)	56.94	50.00	100.00	100.00
PSA-PM(Cosine)	43.06	50.00	100.00	100.00
PSA-PM(Spearman)	56.94	50.00	100.00	100.00

**Figure 7:**
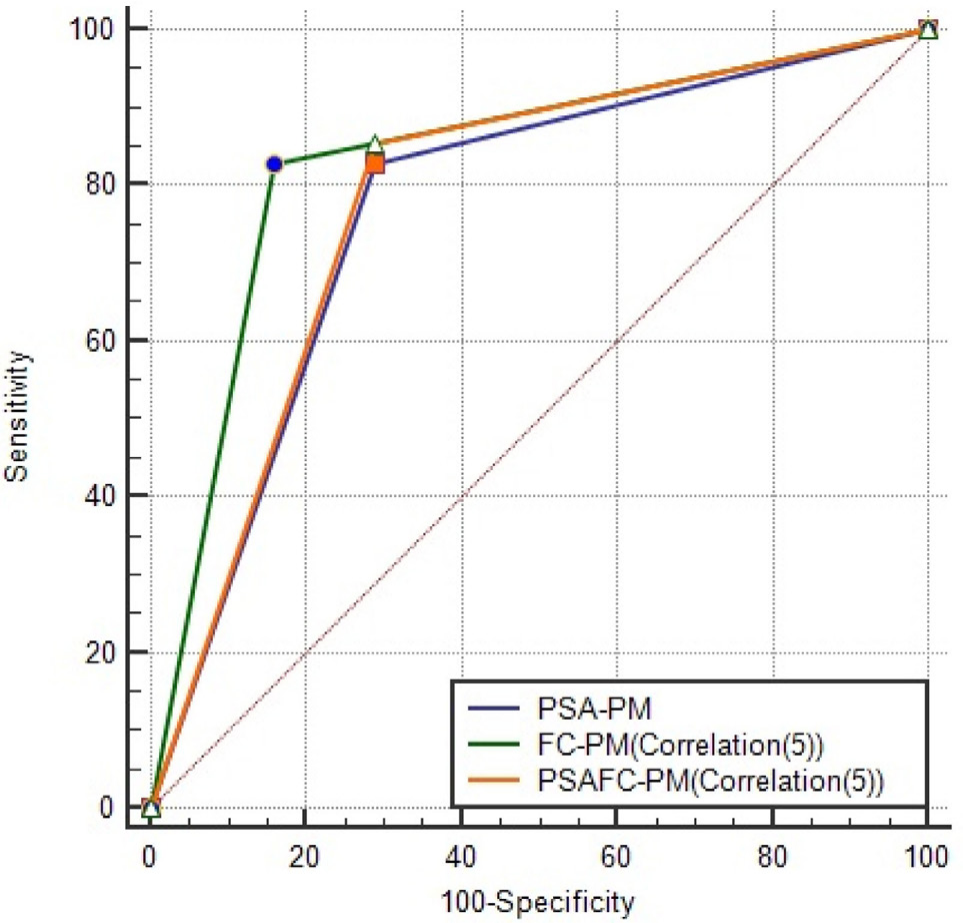
AUCs and optimal ROC points of PSA-PM, FC-PM(Correlation(5)) and PSAFC-PM (Correlation(5)). This figure illustrates the differences among the models in predictive performance. FC-PM (Correlation(5)) was the best model in reducing the false positives. PSA-PM has ORP (TPR = 82.93, FPR = 29.03), FC-PM (Correlation (5)) has ORP (TPR = 82.93, FPR = 16.13), and PSAFC-PM (Correlation (5)) has ORP (TPR = 85.37, FPR = 29.03).

Figure 7 illustrates the AUCs and optimal ROC Points of PSA-PM and FC-PM (Correlation(5)). Table 8 shows a comparison of AUC statistics using PSA-PM and FC-PM. The CI values shown in Table 8 are higher for FC-PM(Correlation(5)) thereby meaning that the model is more capable of achieving higher prediction accuracies. Comparing the classification performances of FC-PM(Correlation(5)) (Accuracy = 83.33%) and the PSA-PM (Accuracy = 77.78%), there is a 5.55% increase in accuracy when using the FC-PM. Furthermore, there is a 12.9% increase in False Positive Rate (FPR) when using PSA-PM, as opposed to when using the FC-PM(Correlation(5)). In conclusion, the FC-PM (Correlation (5)) which is based on immunophenotyping features provides a more accurate identification of prostate cancer than PSA-PM and is better able to discriminate between the presence of benign disease and cancer.

**Table 8:** A comparison using PSA-PM, FC-PM, and PSAFC-PM.

	PSA-PM	FC-PM(Correlation(5))	PSAFC-PM
AUC%	76.95	83.40	78.17
AUC (SE^a^)	0.0590	0.0514	0.0581
AUC 95% CI^b^	0.655–0.861	0.728–0.911	0.669 to 0.870
ORP TPR (%)	82.93	82.93	85.37
ORP FPR (%)	29.03	16.13	29.03
Accuracy (%)	77.78	83.33	79.17
Benign Accuracy (%)	70.79	83.87	70.97
Cancer Accuracy (%)	82.93	82.93	85.37
Misclassified (%)	22.22	16.67	20.83

*^a^Hanley and McNeil (20)*.

*^b^Binomial exact*.

### 3.4 Does Adding the PSA Test Values to the Flow Cytometry Phenotyping Strengthen the Diagnostic Accuracy and Potential?

Given that current clinical practice uses the PSA test as an initial indicator of prostate cancer, we determined whether combining PSA test values with the selected flow cytometry predictors can strengthen diagnostic accuracy of the PSA test (the PSAFC prediction model, PSAFC-PM). The PSA-PM was tuned using the Euclidean distance measure, whereas the PSAFC-PM was tuned with the Correlation distance measure. Although the PSA-PM performed exactly the same when tuned with distance measures other than Euclidean as shown in Table 7, the Euclidean distance measure was selected because it is the simplest to compute. Experiments with various distance measures revealed that PSAFC-PM achieved its highest predictive accuracy using the correlation distance measure. Results of the performance evaluation using the best models are presented in Table 8 and illustrated in Figure 7. Comparing the predictive performance of the PSA-PM to the PSAFC-PM(Correlation(5)), an important observation is that the latter achieved 2.44% higher TPR than the PSA-PM, without increasing the FPR. Furthermore, the PSAFC-PM(Correlation(5)) returned an overall predictive accuracy of 79.17%, whereas the PSA-PM(Correlation(5)) returned 77.78% overall predictive accuracy, and thus an improvement of 1.39% when flow cytometry features were combined with PSA. It is useful to observe the impact of the predictors on the classification accuracy for each group of individuals, i.e., benign disease and cancer. Table 8 holds these values and it also contains the values of FC-PM for comparison purposes. Table 8 shows that the PSAFC-PM(Correlation(5)) performed better than the PSA-PM with regard to identifying benign disease (0.18% improvement), and it was also 2.44% more accurate at identifying cancer than the PSA-PM. In particular, the PSAFC-PM(Correlation(5)) achieved a 85.37% accuracy in detecting cancer, whereas the PSA-PM delivered 82.93% accuracy (a 2.44% difference).

The PSA-based prediction models, PSA-PM and PSAFC-PM, clearly suffer from higher FPRs than the FC-PM model, primarily because combining the PSA with the FC predictors inherits the disadvantage of PSA returning a high number of false positive cases. Table 8 shows that combining PSA with flow cytometry predictors increases the confidence interval and reduces the Standard Error of the AUC (SE) of the prediction compared to using PSA predictors alone, meaning that fewer patients will be misdiagnosed when using the PSAFC-PM, as opposed to the PSA-PM model.

Herein, we propose a predictive model, PSAFC-PM, which improves the diagnostic capacity of the PSA test by combining PSA with flow cytometry features. A very important finding from the experiments is that if current clinical practice favors the continuation of the PSA test as an initial indicator of prostate cancer, then combining PSA predictor with a subset of flow cytometry predictors can increase the accuracy of the initial PSA test.

## 4 Discussion

The results of this study demonstrate that the presence of prostate cancer in asymptomatic men with PSA levels <20 ng ml^−1^ can be better identified using immune cell profiles that have been generated using multiparametric flow cytometricanalysis of the peripheral blood. Prediction models were implemented using an advanced computational data extraction approach and a comprehensive statistical analysis. The computational approach comprised a metaheuristic optimization method, namely the Genetic Algorithm, which identified significant relationships between leukocyte population profiles and the presence of benign disease (no prostate cancer) or prostate cancer. A subset of five flow cytometry features was selected (*CD*8^+^*CD*45*RA*^−^*CD*27^−^*CD*28^−^; *CD*4^+^*CD*45*RA*^−^*CD*27^−^*CD*28^−^; *CD*4^+^*CD*45*RA^+^CD*27^−^*CD*28^−^; *CD*3^−^*CD*19^+^; *CD*3^+^*CD*56^+^*CD*8^+^*CD*4^+^) from a set of 20 features, which could potentially discriminate between the presence of benign disease and prostate cancer. A prostate cancer prediction model was constructed using the selected features and the k-Nearest Neighbor classification algorithm. The proposed model, which takes as input the abovementioned five flow cytometry features, outperformed the predictive model which takes PSA values as input. In particular, the flow cytometry-based model achieved Accuracy = 83.33%, AUC = 83.40%, and optimal ROC points of FPR = 16.13%, TPR = 82.93%, whereas the PSA-based model achieved Accuracy = 77.78%, AUC = 76.95%, and optimal ROC points of FPR = 29.03%, TPR = 82.93%. Combining PSA and flow cytometry-based parameters as predictors achieved Accuracy = 79.17%, AUC = 78.17%, and optimal ROC points of FPR = 29.03% TPR = 85.37%.

Since current clinical practice favors the use of the PSA test as an initial indicator of prostate cancer, complementing the PSA prediction model with a subset of flow cytometry predictions can increase the accuracy of the initial prostate cancer test and reduce the misclassified patient cases. The proposed prediction model has the potential to improve outcomes of prostate cancer patients. Future studies will undertake further evaluations using the identified set of cancer predictors, and explore the use of deep learning algorithms for the analysis and interpretation of high dimensional flow cytometry data.

## 5 Methods

The prediction model was developed using a selected subset of flow cytometry features and the k-Nearest Neighbor (kNN) classification algorithm. The Genetic Algorithm proposed by Ludwig and Nunes (18) was utilized for the feature selection stage, and this algorithm returned the best combination of flow cytometry features (i.e., predictors) for discriminating between patients with benign disease and patients with cancer. These predictors were then input into the kNN classification algorithm. The kNN classifier is used to predict the disease status of an individual using new and previously unseen patient records. Feature selection is important because it enables only the best subset of features (i.e., predictors) to be selected for the prediction task and, thus, removes the “noisy” features that are not useful in identifying cancer.

The Genetic Algorithm is a powerful metaheuristic optimization method which aims to find optimal solutions to NP-hard optimization problems (26)—these are problems which require searching a space for the best solution (27). Let *X* be a *m* x *n* matrix with *m* rows and *n* columns, where *m* is the total number of patient records and *n* is the total number of flow cytometry features. Each patient record, *x_i_*, is represented by an n-dimensional feature vector, and it is given a corresponding known class label *y_i_*, which has a value of either benign disease or cancer. The known labels were derived because of the highly accurate TPTP biopsy. The Genetic Algorithm is designed to take as input the *m* x *n* matrix *X*, and a *m* x 1 vector Y, where each element *y_i_* contains the target output of each patient record. The Genetic Algorithm returns a set of indices of size *λ* containing the selected features. Importantly, the *λ* number of features returned are the best combination of features for discriminating the two groups of individuals (i.e., benign disease or cancer). It was important to use a Genetic Algorithm for the flow cytometry feature selection task for three main reasons:

- There were no significant differences between the mean flow cytometry values of the benign disease and cancer groups (Table 4), as a consequence of which a more sophisticated approach for identifying the best predictor features was needed.
- Searching for the best number of features is a combinatorial optimization problem, such that
  (2) $$\frac{n!}{2(n-\lambda )!},$$
  where *n* is the total number of flow cytometry features and *λ* is the desired number of features. Given that the value of *λ* is not known beforehand, experiments are needed with the number of features starting from *λ* = 2, …, 20. The total possible number of combinations is 104,855,5 making this a computational expensive task, which is also impossible to be completed by basic statistical approaches. The Genetic Algorithm proposed by Ludwig and Nunes (18) was adapted and applied to extract the best set of flow cytometry features.
- When choosing the best subset of features for a predictive modeling task, it is important to take into consideration the interaction between features and the efficiency of these, as a group, for predicting an outcome (i.e., whether a patient belongs to the benign disease or cancer class), as opposed to choosing the best subset of features based on an analysis of each feature alone.

### 5.1 The k-Nearest Neighbor (kNN) Classification Algorithm

The subset of features returned by the Genetic Algorithm was input into the kNN classifier, and this was then used to construct a prediction model based on the particular subset of features. Nearest-neighbor classifiers are based on learning analogy, meaning that by comparing a given test case with training cases that are similar to the test cases. All training cases are represented as points in an n-dimensional space. The kNN classifier is a popular classification method, primarily due to its simplicity. It is a non-parametric approach and, hence, does not make any assumptions about the distribution of the data. When given an unknown case to classify, a kNN classifier searches the pattern space for the *k* training cases, i.e., “nearest neighbors” that are closest to the unknown case (i.e., the case that needs to be classified). Many distance measures exist, including the Euclidean distance, the Minkowski distance, the Hamming distance, Pearson’s correlation coefficient, and cosine similarity. The performance of the kNN classifier depends on the choice of *k*-nearest neighbors, and the distance measure *d* selected. The values selected for *k* and *d* depend on the dataset and the specification of the problem, and for this reason they are selected experimentally. Given a patient record (represented as a data point) *x* holding the flow cytometry values; a *k* number of neighbors; and a distance metric *d*, the kNN classifier first locates the *k* data points (i.e., k patient records) that are the closest to the data point *x* (i.e., patient record *x*) as the k-nearest neighbors to determine the target class of the data point. The proposed kNN approach uses the exhaustive search method, also known as the brute force method. The exhaustive search method finds the distance from each query point (i.e., a record to be classified), *x*, to every point in *X*, ranks them in ascending order, and returns the *k* points with the smallest distances. For the experiments reported in this paper, the kNN classifier can be tuned by selecting a distance measure *d*, and a *k* number of neighbors.

### 5.2 Performance Evaluation Measures

With regard to measuring performance, the aim was to adopt a variety of relevant evaluation metrics in order to get a more representative view of each classifier’s performance. Let |*TP*| be the total number of patients with cancer correctly classified as having cancer; |*TN*| be total the number of benign patients correctly classified as benign; |*FP*| be the total number of benign patients incorrectly classified as cancer patients; |*FN*| be the total number of cancer patients incorrectly classified as benign; |*P*| be the total number of cancer patients that exist in the dataset, where |*P*| = |*TP*| + |*FN*|; and |*N*| be the total number of benign that exist in the dataset, where |*N* | = |*FP*| + |*TN*|. The following commonly used evaluation measures can be defined:

(3) $$\mathrm{Accuracy}=\frac{\left|TP|+|TN\right|}{\left|TP|+|FP|+|FN|+|TN\right|},\in [0,1],$$

(4) $$\mathrm{TPR}=\frac{\left|TP\right|}{\left|TP|+|FN\right|},\in [0,1],$$

(5) $$\mathrm{TNR}=\frac{\left|TN\right|}{\left|TN|+|FP\right|},\in [0,1],$$

(6) $$\mathrm{FNR}=\frac{\left|FN\right|}{\left|TP|+|FN\right|}=1-\mathrm{Sensitivity},\in [0,1],$$

(7) $$\mathrm{FPR}=\frac{\left|FP\right|}{\left|FP|+|TN\right|}=1-\mathrm{Specificity},\in [0,1].$$

The closer the values of Accuracy, True Positive Rate (i.e., TPR, Sensitivity) and True Negative Rate (i.e., TNR, Specificity) are to 1.0, then the better the classification performance of a system. The Receiver Operating Characteristic (ROC) is another important metric which can be used to evaluate the quality of a classifier’s performance. The optimal operating point of the ROC curve is made up of the False Positive Rate (FPR) and True Positive Rate (TPR) values. The optimal operating point for the ROC curve is computed by finding the slope, *S*, using function (8) and then identifying the optimal operating point by moving the straight line with slope *S* from the upper left corner of the ROC plot (*FPR* = 0, *TPR* = 1) down and to the right, until it intersects the ROC curve.

(8) $$S=\frac{\mathrm{Cost}(P|N)-\mathrm{Cost}(N|N)}{\mathrm{Cost}(N|P)-\mathrm{Cost}(P|P)}\times \frac{N}{P},$$

where *Cost*(*N*|*P*) is the cost of misclassifying a positive class as a negative class; *Cost*(*P*|*N*) is the cost of misclassifying a negative class, as a positive class; *P* and *N* are the total instance counts in the positive and negative class, respectively. The Area Under the ROC Curve (AUC) can be computed and reflects a system’s performance at discriminating between the data obtained from individuals with benign disease and patients with cancer. The larger the AUC, the better the overall capacity of the classification system to correctly identify benign disease and cancer.

## 6 Potential Impact

It is essential that men with low-risk prostate abnormalities are not diagnosed as having prostate cancer, as even those with low-grade disease do not require active treatment, yet they become “labeled” as having cancer. This can have adverse psychological and financial consequences and assign these men to life-long surveillance. The strategies described herein have the potential to deliver new approaches for diagnosing asymptomatic men with an elevated PSA <20 ng l^−1^. Inserting the data derived from the analysis of the peripheral blood from an individual into the algorithm will return a prediction about that individual. The algorithm could be retrained when more patient data are collected in order to learn patterns from a larger population, and it is possible that this will increase the accuracy of the approach. For example, re-training can occur every 50 new records. Such approaches will spare men with benign disease or low-risk cancer from unnecessary invasive diagnostic procedures such as TRUS guided prostate biopsies or TPTPB.

## Ethics Statement

Research Protocols were registered and approved by the National Research Ethics Service (NRES) Committee East Midlands and by the Research and Development Department in the University Hospitals of Leicester NHS Trust. All participants were given information sheets explaining the nature of the study and all provided informed consent. All samples were collected by suitably qualified individuals using standard procedures. Ethical approval for the collection and use of samples from the TPTPB cohort (Project Title: Defining the role of Transperineal Template-guided prostate biopsy) was given by NRES Committee East Midlands-Derby 1 (NREC Reference number: 11/EM/3012; UHL11068). Ethical approval for the collection of peripheral blood from healthy volunteers was obtained from the Nottingham Trent University College of Science and Technology Human Ethics Committee (Application numbers 165 and 412).

## Author Contributions

GC computationally analyzed the flow cytometry data, prepared and tested the algorithms, analyzed the results, wrote the first draft, and made a significant contribution to the preparation of the manuscript. SM contributed to the preparation, staining and analysis of the flow cytometry data, and generated the multidimensional flow cytometry datasets on which the study has been based. SR, GF, and SH contributed to the preparation, staining, and analysis of the flow cytometry data, and generated the multidimensional flow cytometry datasets on which the study has been based. MK identified the clinical need, provided access to clinical samples and clinical data, and made a significant contribution to the preparation of the manuscript. AP conceived the study and made a significant contribution to the interpretation of the data and the preparation of the manuscript. All authors reviewed the manuscript.

## Conflict of Interest Statement

The authors declare that the research was conducted in the absence of any commercial or financial relationships that could be construed as a potential conflict of interest.
